# Supplementary material for: Nanopore sequencing and the Shasta toolkit enable efficient de novo assembly of eleven human genomes
Source: Nat Biotechnol. 2020 May 4;38(9):1044–53. doi: 10.1038/s41587-020-0503-6 (PMC7483855; doi:10.1038/s41587-020-0503-6)
Supplement: Supplementary file 1 — Supplementary Notes, Figs. 1–8 and Tables 1–36. [file 41587_2020_503_MOESM1_ESM.pdf]

nature biotechnology

SUPPLEMENTARY INFORMATION

https://doi.org/10.1038/s41587-020-0503-6

In the format provided by the authors and unedited.

Nanopore sequencing and the Shasta toolkit enable efficient de novo assembly of eleven human genomes

Kishwar Shafin[1,11], Trevor Pesout[1,11], Ryan Lorig-Roach[1,11], Marina Haukness[1,11], Hugh E. Olsen[1,11], Colleen Bosworth[1], Joel Armstrong[1], Kristof Tigyi[1,2], Nicholas Maurer[1], Sergey Koren[3], Fritz J. Sedlazeck[4], Tobias Marschall[5], Simon Mayes[6], Vania Costa[6], Justin M. Zook[7], Kelvin J. Liu[8], Duncan Kilburn[8], Melanie Sorensen[9], Katy M. Munson[9], Mitchell R. Vollger[9], Jean Monlong[1], Erik Garrison[1], Evan E. Eichler[2,9], Sofie Salama[1,2], David Haussler[1,2], Richard E. Green[1], Mark Akeson[1], Adam Phillippy[3], Karen H. Miga[1], Paolo Carnevali[10] ✉, Miten Jain[1] ✉ and Benedict Paten[1] ✉

[1]UC Santa Cruz Genomics Institute, Santa Cruz, CA, USA. [2]Howard Hughes Medical Institute, University of California, Santa Cruz, CA, USA. [3]Genome Informatics Section, Computational and Statistical Genomics Branch, National Human Genome Research Institute, Bethesda, MD, USA. [4]Baylor College of Medicine, Human Genome Sequencing Center, Houston, TX, USA. [5]Max Planck Institute for Informatics, Saarbrücken, Germany. [6]Oxford Nanopore Technologies, Oxford, UK. [7]National Institute of Standards and Technology, Gaithersburg, MD, USA. [8]Circulomics Inc., Baltimore, MD, USA. [9]Department of Genome Sciences, University of Washington School of Medicine, Seattle, WA, USA. [10]Chan Zuckerberg Initiative, Redwood City, CA, USA. [11]These authors contributed equally: Kishwar Shafin, Trevor Pesout, Ryan Lorig-Roach, Marina Haukness, Hugh E. Olsen. ✉e-mail: paolo@chanzuckerberg.com; miten@soe.ucsc.edu; bpaten@ucsc.edu

Supplementary Results: Nanopore sequencing and the Shasta toolkit enable efficient *de novo* assembly of eleven human genomes

Supplementary Notes

Execution Parameters

Shasta

All Shasta runs used Shasta version 0.1.0 built from `https://github.com/chanzuckerberg/shasta`. Rather than using the distributed version of the release, the source code was rebuilt locally for best performance as recommended by Shasta documentation.

The Shasta executable was run with the following command:

```
shasta \
    --memoryMode filesystem \
    --memoryBacking 2M
```

Canu

Canu 1.8 from `https://github.com/marbl/canu` was run with the following command:

```
canu \
    -p asm \
    -d asm \
    genomeSize=3.1g \
    'corMhapOptions=--threshold 0.8 --num-hashes 512
               --ordered-sketch-size 1000 --ordered-kmer-size 14' \
    'gridOptionsJobName=mom' \
    'gridOptions=--time=240:00:00 --partition=norm' \
    'stageDirectory=/lscratch/$SLURM_JOBID' \
    'gridEngineStageOption=--gres=lscratch:100' \
    'correctedErrorRate=0.105' \
    -nanopore-raw input.fastq.gz
```

Wtdbg2

Wtdbg2 version 2.3 from `https://github.com/ruanjue/wtdbg2` was run with the following commands:

```
wtdbg2 \
    -t 0 \
    -x ont \
    -L 10000 \
    -g 3.3g \
    -i reads1.fastq.gz \
    -i reads2.fastq.gz \
```

```
    -i reads3.fastq.gz \
    -o wtdbg2-assembly

wtpoa-cns \
    -t 31 \
    -i wtdbg2-assembly.ctg.lay.gz \
    -f \
    -o wtdbg2-assembly.fa
```

Flye

Flye version 2.4.2 from `https://github.com/fenderglass/Flye` was run with the following command:

```
flye \
    --nano-raw reads1.10kb.fastq.gz reads2.10kb.fastq.gz reads3.10kb.fastq.gz \
    --genome-size 3.3g \
    --out-dir flye \
    --threads 123
```

Racon

We used a home-grown script to manage running 4 iterations of Racon, v1.3.2. The code for the script can be found here https://github.com/rlorigro/nanopore_assembly_and_polishing_assessment, and was run with the following command:

```
python3 /home/ubuntu/software/nanopore_assembly_and_polishing_assessment/polish.py \
    --true_ref hg38.fa \
    --contigs assembly.fasta \
    --sequences reads.fasta \
    --output_dir racon \
    --n_passes 4
```

When run for the analysis to produce Supplemental Table 26, the `n_passes` parameter was set to 1.

Medaka

Medaka version 0.6.0-alpha.3 from `https://github.com/nanoporetech/medaka` was run with the following commands:

```
medaka consensus \
    -i reads5.fasta \
    -d assembly_racon4x.fasta \
    -o medaka \
    -t 64 \
    -m r941_flip235

medaka stitch \
    medaka/consensus_probs.hdf \
    medaka/consensus.fasta
```

No changes in the arguments were used for the analysis that produced Supplemental Table 26. This includes the GPU mode, which is configured during compilation.

Minialign

Minialign is bundled with Medaka, and was run with the following commands:

```
mini_align \
    -i reads.fasta \
    -r assembly.fasta \
    -P \
    -m \
    -p medaka/calls_to_draft \
```

```
    -t 60
```

Minimap2, Samtools

Minimap2 version 2.15-r908-dirty from `https://github.com/lh3/minimap2`. We used samtools 1.7 using htslib 1.7-2 for sorting and filtering. The following three commands were piped into each other:

```
minimap2 \
    -ax map-ont \
    -t 70 \
    assembly.fasta \
    reads.fasta

samtools sort \
    -@ 70

samtools view \
    -hb \
    -F 0x104 \
    >align.bam
```

MarginPolish

MarginPolish 1.0.0 was compiled from `https://github.com/UCSC-nanopore-cgl/MarginPolish` run with the following command:

```
marginPolish \
    input.bam \
    input.fa \
    allParams.np.human.guppy-ff-235.json \
    -f \
    -o output\_location \
    -t 70
```

When run to produce Supplemental Table 26, MarginPolish was used compiled from the commit `4c1da1e1b3efc739e9c48913416efac619d3d40c` on GitHub.

HELEN

HELEN version 0.1 from `https://github.com/kishwarshafin/helen` was run with the following commands:

```
python3 /home/ubuntu/software/helen/call_consensus.py \
    -i images/ \
    -b 1024 \
    -w 16 \
    -t 32 \
    -m r941_flip235_v001.pkl \
    -o out \
    -g

python3 /home/ubuntu/software/helen/stitch.py \
    -i out/helen_predictions_05312019_183902.hdf \
    -o out/ \
    -p polished_assembly \
    -t 32
```

HiRise

HiRise was run via a docker container, with access given by Dovetail Genomics. The HiRise version was v2.1.6, with the HiRise Helper version 2.1.10 and the HiRise Utils version v2.1.7-3-g98c1a1b. Default parameters were used.

Long Ranger

The 10X Long Ranger Align pipeline (v2.2) was used for any alignment of 10X reads to a reference. An example sequence of commands was:

```
longranger mkref assembly.fa

longranger align \
    --id 10x-chm13-chrX-round1 \
    --reference refdata-assembly \
    --fastqs fastq/
```

Pilon

An example Pilon command (using v1.23) is below:

```
java -Xmx200G -jar pilon-1.23.jar \
    --bam align.bam \
    --genome assembly.fa \
    --threads 32  \
    --output pilon-out
```

Trio-binning

For HG00733, the parental read sample accessions were obtained from 1000 genome database:

```
http://www.internationalgenome.org/data-portal/sample/HG00731
http://www.internationalgenome.org/data-portal/sample/HG00732
```

Briefly, k-mers were counted with `meryl`, subtracted to generate maternal/paternal sets, and any k-mers occurring less than 6 times for maternal k-mers and 5 times for paternal k-mers were not used. Binning did not use normalization by k-mer set size. This resulted in 35.2x maternal, 37.3x paternal, and 5.6x unclassified. Assembly did not use the unclassified reads and ran with the command:

```
canu \
    -p asm \
    -d <mom/dad>
    'genomeSize=3.1g' \
    'corMhapOptions=--threshold 0.8 --num-hashes 512
                --ordered-sketch-size 1000 --ordered-kmer-size 14' \
    'corMinCoverage=0'
```

Each haplotype assembly required approximately 100k CPU hours (4-5 days). A subsequent run using Canu 1.8 and automated binning with the command:

```
canu \
    -p asm \
    -d asm \
    'genomeSize=3.1g' \
    'corMhapOptions=--threshold 0.8 --num-hashes 512
                --ordered-sketch-size 1000 --ordered-kmer-size 14' \
    'gridOptionsJobName=733_trio' \
    'corMinCoverage=0' \
    -haplotypeMOM hg0732/*fastq.gz \
    -haplotypeDAD hg0731/*.fastq.gz
```

resulted in a similar classification split (35.1x dad, 36.7x mom, 5.6x unknown) and assembly (manual: dad=16.6 NG50, mom=18.1 NG50; automated: dad=14.1 NG50, mom=19.9 NG50).

For HG0002, illumina data for the parents was downloaded from the GIAB ftp site:

```
ftp://ftp-trace.ncbi.nlm.nih.gov/giab/ftp/data/AshkenazimTrio/HG003_NA24149_father \
    /NIST_HiSeq_HG003_Homogeneity-12389378/HG003_HiSeq300x_fastq/
ftp://ftp-trace.ncbi.nlm.nih.gov/giab/ftp/data/AshkenazimTrio/HG004_NA24143_mother \
    /NIST_HiSeq_HG004_Homogeneity-14572558/HG004_HiSeq300x_fastq/
```

K-mers were counted as before, subtracted, and filtered to exclude k-mers occuring less than 25 times in the maternal or paternal set. The classification resulted in 24x maternal, 23x paternal, and 3.5x unknown. Only classified reads were used for assembly with the command:

```
canu \
    -p asm \
    -d <mom/dad> \
    'genomeSize=3.1g' \
    'corMhapOptions=--threshold 0.8 --num-hashes 512
               --ordered-sketch-size 1000 --ordered-kmer-size 14'  \
    'corMinCoverage=0'
```

Each haplotype assembly required approximately 100k cpu hours (4-5 days).

QUAST

When using QUAST to evaluate assembly statistics and run BUSCO, we used the following command below. `--large` indicates that the genome is large, `--fragmented` indicates the reference genome may be fragmented, `--min-identity 80` indicates that alignments with identity less than 80% will be filtered, `--conserved-genes-finding` indicates that BUSCO will be run to find universal single-copy orthologs, and `eukaryote` indicates that the genome is from a eukaryote.

```
quast-lg.py \
    --threads 12 \
    -r truth_assembly.fa \
    -o quast-out \
    --large \
    --min-identity 80 \
    --fragmented \
    --conserved-genes-finding \
    --eukaryote \
    assembly.fa
```

Benchmarking assemblies using Pomoxis

The truth assembly files and the reported error-rates are described in Online methods.

To benchmark the assemblies, we used `assess_assembly` pull 37 from Pomoxis (https://github.com/nanoporetech/pomoxis/pull/37). This tool is developed and suggested by the research group of Oxford Nanopore Technology. We added the functionality to ignore large insertions and deletions. The installation instruction of Pomoxis can be found on the github page https://github.com/nanoporetech/pomoxis. The parameters we used are:

- -i: The input assembly (fasta).
- -r: The reference fasta file. (The truth assembly)
- -b: Bed file containing reference regions to assess.
- -p: Prefix of the output file names.
- -c: Chunk size. Input reads/contigs will be broken into chunks prior to alignment.
- -t: Number of threads to use.
- -T: Trim consensus to primary alignments of truth to assembly.
- -l: Ignore insertions and deletions longer than this value, 0 means include everything. (default 0)

We compared the HG002 samples, we gathered the truth assembly `hg002_truth_assembly.fa`, a bed file `hg002_confident.bed` describing the confident regions and a shasta assembly `hg002_shasta_assembly.fa` and ran the following command.

```
assess_assembly \
    -i hg002_shasta_assembly.fa \
    -r hg002_truth_assembly.fa \
```

```
    -b hg002_confident.bed \
    -p hg002_shasta_assessment \
    -c 1000 \
    -l 50 \
    -t 32 \
    -T
```

In this setup, the `assess_assembly` module computes the error rate of the input `hg002_shasta_assembly.fa` that aligns to the high-confidence region defined in the `hg002_confident.bed` of `hg002_truth_assembly.fa` assembly. Also, the `-T` parameter limits the assessment to regions where there is an alignment between the truth and the input assembly.

For HG00733 sample, we used the high-quality phased PacBio assembly. We got `hg00733_truth_assembly.fa` and the `hg00733_shasta_assembly.fa` and ran the following command for assessment.

```
assess_assembly \
    -i hg00733_shasta_assembly.fa \
    -r hg00733_truth_assembly.fa \
    -p hg00733_shasta_assessment \
    -t 32 \
    -c 1000 \
    -l 50 \
    -T
```

As the truth assembly of HG00733 does not define any high-confidence region, we do a whole genome comparison where there is an alignment between the truth and the input assembly enforced by the `-T` parameter. For CHM13 and all other assemblies, we used the same command as HG00733. The output of this program reports different error rates described in the online methods section.

Extracting common assembly regions

To create a bed file describing the regions where all the assemblers have an assembly, we used `mini_align` available https://github.com/nanoporetech/pomoxis/, and `bedtools` which can be found in https://bedtools.readthedocs.io/en/latest/.

We first align the assembly to the truth assembly using `mini_align`.

```
mini_align -P -m -c 100000 \
    -r truth_assembly.fa \
    -i assembler_assembly.fa \
    -t 64 \
    -p assembler_2_truth
```

Then we extract the regions where the assemblers have an assembly:

```
bedtools bamtobed -i assembler_2_truth.bam > assembler.bed
```

Finally we do an intersection of all the bed files that we get from each assemblers. For HG002, we also included the high confidence region bed file.

```
multiIntersectBed -i <list_of_bed> | awk '$4 == <number_of_beds>' > common_regions.bed

sort -k1,1 -k2,2n common_regions_between_assemblers_hg002.bed > common_regions.sort.bed

bedtools merge -i common_regions.sort.bed > common_regions_between_assemblers.bed
```

Extracting chrX from assemblies

To analyze subsets of the CHM13 assemblies which correspond to regions in chrX, we used the following steps to extract contigs. Briefly, we align the assembly to GRCh38, identify any assembly contig which had a primary or supplementary alignment to chrX, and extract these segments.

```
minimap2 -ax asm20 -t 32 GRCh38.fa assembly.fa | samtools view -hb >unsorted.bam
samtools sort -@ 32 unsorted.bam | samtools view -hb >assembly.bam
samtools index -@ 32 assembly.bam
```

```
samtools view -F 0x104 assembly.bam chrX | awk '{print $1}' | sort | uniq >segments.txt
extract_fasta_segments.py -i assembly.fa -s segments.txt -o assembly.hg38_chrX.fa
```

The script `extract_fasta_segments.py` can be found at `https://github.com/tpesout/genomics_scripts`.

Supplementary Results

Nanopore sequencing eleven human genomes in nine days

Supplementary Table 1: Read N50s stratified by sample and flowcell (three for each sample) for 11 samples.

Sample	Flowcell No.	Flowcell N50	Sample N50
GM24143	1	48891	46757
	2	47044	
	3	44335	
GM24149	1	46054	43306
	2	44245	
	3	39618	
GM24385	1	50349	48705
	2	49319	
	3	46448	
HG00733	1	29862	29584
	2	30473	
	3	28417	
HG01109	1	48795	45894
	2	44218	
	3	44670	
HG01243	1	45467	43567
	2	44681	
	3	40554	
HG02055	1	44320	45457
	2	47148	
	3	44902	
HG02080	1	38519	39319
	2	40123	
	3	39315	
HG02723	1	50509	49723
	2	47842	
	3	50817	
HG03098	1	41463	40629
	2	42308	
	3	38115	
HG03492	1	32149	30168
	2	30063	
	3	28292	
**Average**	**-**	**41889**	**42101**

Supplementary Table 2: Throughput stratified by sample and flowcell (three for each sample) in gigabases (Gb) for 11 samples.

Sample	Flowcell No.	Flowcell (Gb)	Sample (Gb)	Coverage
GM24143	1	87	280	84.72
	2	97		
	3	95		
GM24149	1	82	273	82.6
	2	107		
	3	84		
GM24385	1	26	157	47.43
	2	71		
	3	59		
HG00733	1	62	242	73.45
	2	90		
	3	89		
HG01109	1	71	219	66.48
	2	79		
	3	70		
HG01243	1	71	187	56.68
	2	73		
	3	43		
HG02055	1	71	202	61.33
	2	67		
	3	65		
HG02080	1	71	172	52.21
	2	42		
	3	59		
HG02723	1	81	227	68.7
	2	69		
	3	78		
HG03098	1	79	177	53.63
	2	40		
	3	58		
HG03492	1	61	158	47.74
	2	45		
	3	51		
**Average**	**-**	**69**	**208**	**63.18**

Supplementary Table 3: Mean, median, and modal values for read alignment identities of 11 samples, aligned to GRCh38. Metrics were generated per read. Total gigabases of read data for each sample are detailed in Supplementary Table 2

Sample	Mean	Median	Mode
GM24143	0.87188	0.89651	0.920
GM24149	0.87665	0.90511	0.930
GM24385	0.88276	0.91143	0.935
HG00733	0.87165	0.89682	0.925
HG01109	0.87033	0.89845	0.930
HG01243	0.88525	0.91435	0.935
HG02055	0.87215	0.90572	0.930
HG02080	0.88188	0.91259	0.935
HG02723	0.84914	0.87565	0.920
HG03098	0.85522	0.88156	0.915
**All samples:**	**0.87251**	**0.90068**	**0.930**

Supplementary Table 4: Summary read statistics derived from human saliva sequencing.

Reads	Bases	Mean Length	Median Length	Read N50
594,753	10,961,203,887	18,430	15,580	27,778

Shasta: assembling a human genome from nanopore reads in under 6 hours

Supplementary Table 5: QUAST assembly metrics of three samples on four assemblers before polishing, compared against GRCh38 with no alternate contigs.

Sample	Metric	Shasta	Wtdbg2	Flye	Canu
HG00733	# contigs	2,150	5,086	1,852	778
	Total length	2,783,599,890	2,792,376,827	2,816,034,584	2,900,719,051
	N50	24,429,871	18,763,119	28,763,002	44,759,083
	NG50	21,088,309	15,338,021	25,227,330	40,627,903
	# disagreements	814	3,985	6,555	4,570
	Genome fraction (%)	94.982	92.938	95.763	96.404
	Duplication ratio	0.995	1.005	0.986	1.014
	# mismatches per 100 kbp	156.21	248.78	506.12	231.24
	# indels per 100 kbp	453.97	664.90	1,480.91	677.26
	Total aligned length	2,775,307,347	2,742,343,142	2,769,440,009	2,858,769,830
	NA50	16,052,981	9,106,500	18,577,806	21,157,324
	NGA50	12,765,264	7,787,949	16,267,214	19,945,150
HG002	# contigs	1,847	5,310	1,627	767
	Total length	2,801,200,983	2,793,889,694	2,819,241,152	2,901,099,163
	N50	23,346,484	15,380,722	31,253,170	33,064,788
	NG50	20,205,529	13,750,884	25,917,293	32,340,595
	# disagreements	901	3,572	5,881	3,882
	Genome fraction (%)	95.622	93.136	96.228	96.959
	Duplication ratio	0.995	1.004	0.981	1.009
	# mismatches per 100 kbp	167.75	261.72	549.10	231.39
	# indels per 100 kbp	520.33	796.71	1,650.63	792.45
	Total aligned length	2,792,458,737	2,743,401,414	2,768,347,339	2,863,787,213
	NA50	16,068,951	8,564,600	18,803,788	21,330,391
	NGA50	14,189,972	7,361,363	16,079,132	18,175,258
CHM13	# contigs	1,236	6,428	1,269	558
	Total length	2,809,087,051	2,836,802,421	2,857,931,691	2,919,690,848
	N50	46,037,322	15,522,332	36,829,446	80,507,947
	NG50	41,091,906	14,039,241	35,319,460	79,504,166
	# disagreements	1,051	4,202	5,452	4,768
	Genome fraction (%)	95.307	93.124	96.022	96.553
	Duplication ratio	1.000	1.017	0.997	1.014
	# mismatches per 100 kbp	155.15	256.17	443.85	226.04
	# indels per 100 kbp	358.45	535.46	1,023.79	484.46
	Total aligned length	2,798,043,587	2,780,449,715	2,807,157,420	2,864,418,837
	NA50	23,475,255	6,786,237	18,991,999	25,611,947
	NGA50	18,990,051	5,892,796	17,032,972	23,819,455

Supplementary Table 6: QUAST disagreement count for four assemblers on different regions of the genome for four samples. We report disagreements that happen in all chromosomes of GRCh38, then incrementally exclude centromeric regions, segmental duplication regions (Seg Dups), and all other regions enriched for SVs (chrY, acrocentric chromosome arms, and QH-regions)

Sample	Assembler	Disagreements in GRCh38 autosomes and chrX, chrY	Disagreements outside centromeres	Disagreements outside centromeres and seg dups	Disagreements outside centromeres, seg dups, chrY, acrocentric chr arms, and QH-regions
HG002	Shasta	901	755	284	121
	Flye	5881	1226	513	117
	Canu	3882	2347	689	216
	Wtdbg2	3572	1213	484	148
HG00733	Shasta	814	662	256	110
	Flye	6555	1261	604	134
	Canu	4570	2791	755	224
	Wtdbg2	3985	1166	474	135
CHM13	Shasta	1051	795	333	129
	Flye	5452	1228	448	107
	Canu	4768	2764	864	164
	Wtdbg2	4202	1519	592	249

Supplementary Table 7: Disagreement count in the intersection of the assemblies for each sample (see Online Methods). Total Disagreements describes all disagreements found in 100bp windows before taking the intersection; note that these counts are very close to those reported by QUAST. Consensus Disagreements describes disagreements in the intersection of the four assemblies. Genome fraction describes total coverage over GRCh38 for the consensus sequence.

Sample	Assembler	Total Disagreements	Consensus Disagreements	Genome Fraction
HG002	Shasta	863	179	87.16%
	Flye	5823	178	87.16%
	Canu	3779	328	87.16%
	Wtdbg2	3509	215	87.16%
HG00733	Shasta	792	161	87.43%
	Flye	6546	178	87.43%
	Canu	4524	383	87.43%
	Wtdbg2	3975	205	87.43%
CHM13	Shasta	1033	242	87.53%
	Flye	5446	217	87.53%
	Canu	4682	712	87.53%
	Wtdbg2	4190	404	87.53%

Supplementary Table 8: Disagreement count and fraction of genome covered on chromosome X for four assemblers on CHM13 assemblies with no polishing, compared to the chromosome X assembly from the Telomere-to-Telomere Consortium. These numbers were obtained via running QUAST.

Assembler	Disagreements	Genome Fraction
Shasta	5	97.73%
Wtdbg2	87	94.17%
Flye	18	98.41%
Canu	9	98.16%

Supplementary Table 9: BAC analysis on selected dataset. BACs were selected (31 of CHM13 and 16 of HG00733) for falling within unique regions of the genome, specifically >10 Kb away from the closest segmental duplication. *Closed* refers to the number of BACs for which 99.5% of their length aligns to a single locus in the assembly. *Attempted* refers to the number of BACs which have an alignment for >5 Kb of sequence with >90% identity to only one contig (BACs which have such alignments to multiple contigs are excluded). Identity metrics are for *closed* BACs.

Sample	Assembler	BAC counts				Median Quality		Mean Quality	
		Total	Attempted	Closed	Closed of attempted %	Identity %	QV	Identity %	QV
CHM13	Canu	31	31	30	96.77	99.40	22.18	99.34	21.84
	Flye	31	31	31	100.00	97.58	16.17	97.65	16.28
	Shasta	31	31	31	100.00	99.55	23.51	99.51	23.07
	Wtdbg2	31	29	28	96.55	99.46	22.71	99.39	22.15
HG00733	Canu	16	16	15	93.75	98.74	18.98	98.61	18.56
	Flye	16	16	16	100	97.99	16.97	98.01	17.02
	Shasta	16	16	16	100	98.84	19.38	98.79	19.20
	Wtdbg2	16	16	16	100	98.81	19.26	98.79	19.20

Supplementary Table 10: BAC analysis on full dataset, 341 on CHM13 and 179 on HG00733. *Closed* refers to the number of BACs for which 99.5% of their length aligns to a single locus. *Attempted* refers to the number of BACs which have an alignment for >5Kb of sequence with >90% identity to only one contig (BACs which have such alignments to multiple contigs are excluded). Identity metrics are for *closed* BACs.

Sample	Assembler Polisher	BAC counts				Median Quality		Mean Quality	
		Total	Attempted	Closed	Closed of attempted %	Identity %	QV	Identity %	QV
CHM13	Canu	341	309	287	92.88	99.22	21.07	98.93	19.7
	Flye	341	227	202	88.98	97.54	16.09	97.51	16.03
	Shasta	341	94	92	97.87	99.47	22.74	99.37	21.99
	Wtdbg2	341	70	62	88.57	99.36	21.96	99.28	21.43
HG00733	Canu	179	137	124	90.51	98.73	18.95	98.43	18.05
	Flye	179	98	80	81.63	98.09	17.18	97.76	16.49
	Shasta	179	42	40	95.23	98.76	19.08	98.13	17.30
	Wtdbg2	179	52	46	88.46	98.70	18.87	98.02	17.04

Supplementary Table 11: BAC analysis intersection of attemted BACs by all four assemblers, 65 on CHM13 and 27 on HG00733. *Closed* refers to the number of BACs for which 99.5% of their length aligns to a single locus. *Attempted* refers to the number of BACs which have an alignment for >5Kb of sequence with >90% identity to only one contig (BACs which have such alignments to multiple contigs are excluded). Identity metrics are for *closed* BACs.

Sample	Assembler Polisher	BAC counts				Median Quality		Mean Quality	
		Total	Attempted	Closed	Closed of attempted %	Identity %	QV	Identity %	QV
CHM13	Canu	65	65	64	98.50	99.29	21.53	99.21	21.01
	Flye	65	65	65	100.00	97.57	16.16	97.61	16.22
	Shasta	65	65	65	100.00	99.50	23.03	99.41	22.33
	Wtdbg2	65	65	59	90.80	99.39	22.17	99.29	21.49
HG00733	Canu	27	27	26	96.30	98.66	18.76	98.54	18.37
	Flye	27	27	27	100.00	98.07	17.14	98.08	17.16
	Shasta	27	27	27	100.00	98.80	19.23	98.30	17.71
	Wtdbg2	27	27	26	96.30	98.75	19.01	98.53	18.32

Supplementary Table 12: Base-level accuracies on four different assemblers for three samples. Analysis is performed with whole-genome truth sequences.

Sample	Assembler	Percentage Errors			
		Balanced	Identity	Deletion	Insertion
HG002 Guppy 2.3.5	Shasta	0.975%	0.061%	0.849%	0.065%
	Wtdbg2	1.181%	0.080%	1.073%	0.029%
	Canu	1.400%	0.065%	1.316%	0.020%
	Flye	1.636%	0.068%	0.450%	1.118%
HG00733 Guppy 2.3.5	Shasta	1.062%	0.083%	0.887%	0.093%
	Wtdbg2	1.217%	0.108%	1.059%	0.051%
	Canu	1.328%	0.074%	1.224%	0.031%
	Flye	1.854%	0.089%	0.445%	1.320%
CHM13 Guppy 2.3.1	Shasta	0.540%	0.039%	0.430%	0.072%
	Wtdbg2	0.689%	0.068%	0.583%	0.038%
	Canu	0.705%	0.038%	0.643%	0.024%
	Flye	2.213%	0.051%	0.448%	1.715%

Supplementary Table 13: Base-level accuracies on four different assemblers for three samples in the regions of intersection of the assemblies. Analysis is performed only on regions where all assemblers have an assembled sequence.

Sample	Assembler	Percentage Errors			
		Balanced	Identity	Deletion	Insertion
HG002 Guppy 2.3.5	Shasta	0.943%	0.056%	0.823%	0.064%
	Wtdbg2	1.145%	0.077%	1.041%	0.028%
	Canu	1.319%	0.050%	1.253%	0.016%
	Flye	1.554%	0.063%	0.432%	1.059%
HG00733 Guppy 2.3.5	Shasta	1.021%	0.064%	0.875%	0.083%
	Wtdbg2	1.162%	0.088%	1.034%	0.041%
	Canu	1.307%	0.065%	1.213%	0.030%
	Flye	1.847%	0.068%	0.431%	1.348%
CHM13 Guppy 2.3.1	Shasta	0.513%	0.016%	0.406%	0.048%
	Wtdbg2	0.660%	0.054%	0.575%	0.030%
	Canu	0.692%	0.027%	0.645%	0.021%
	Flye	2.198%	0.036%	0.460%	1.702%

Supplementary Table 14: Runtime and cost of three assembly workflows on Amazon Web Services (AWS) platform.

Method	Sample	Minutes	Threads Used	Peak Memory	AWS Instance Type	AWS Instance Cost
WTDBG2	HG00733	2971	63	365	r5a.16xlarge	$3.62
	GM24385	1752	63	293	r5a.16xlarge	$3.62
	CHM13	1655	63	312	r5a.16xlarge	$3.62
WTDBG2 (wtpoa-cns)	HG00733	248	31	12	r5a.16xlarge	$3.62
	GM24385	274	24	12	r5a.16xlarge	$3.62
	CHM13	257	31	12	r5a.16xlarge	$3.62
Flye	HG00733	3421	123	1013	x1.32xlarge	$13.34
	GM24385	3749	64	727	x1.16xlarge	$6.67
	CHM13	4084	126	911	x1.32xlarge	$13.34
Shasta	HG00733	298	128	966	x1.32xlarge	$13.34
	HG01109	355	128	-	x1.32xlarge	$13.34
	HG01243	296	128	-	x1.32xlarge	$13.34
	HG02055	309	128	-	x1.32xlarge	$13.34
	HG02080	276	128	-	x1.32xlarge	$13.34
	HG02723	373	128	-	x1.32xlarge	$13.34
	HG03098	238	128	-	x1.32xlarge	$13.34
	HG03492	200	128	-	x1.32xlarge	$13.34
	GM24385	240	128	692	x1.32xlarge	$13.34
	GM24149	427	128	-	x1.32xlarge	$13.34
	GM24143	451	128	-	x1.32xlarge	$13.34
	CHM13	317	128	-	x1.32xlarge	$13.34

Supplementary Table 15: Runtime breakdown for each step of the Shasta assembler.

Sample	Input	MinHash	Alignments	Marker graph creation	Transitive reduction	Assemble	Output	Other	Total
HG00733	30	9	93	73	17	15	2	55	298
HG01109	29	10	136	89	16	17	2	53	355
HG01243	23	7	104	73	16	15	2	51	296
HG02055	25	9	113	73	15	15	2	53	309
HG02080	22	7	95	67	15	14	2	49	276
HG02723	29	9	146	89	19	16	2	59	373
HG03098	23	8	73	53	14	14	2	47	238
HG03492	19	7	57	44	11	14	2	40	200
GM24385	20	7	92	49	12	13	2	41	240
GM24149	34	11	149	124	21	18	2	64	427
GM24143	35	11	168	120	24	18	2	69	451
CHM13	21	6	173	67	12	13	2	46	345
Average	26	8	117	77	16	15	2	52	317
Percent of total	8%	3%	37%	24%	5%	5%	1%	17%	100%

Supplementary Table 16: Structural variants extracted from HG002 assembly graph compared to GIAB SV set in high-confidence regions.

Metric	HG002					
	TP	FP	FN	Precision	Recall	F_1
Total	2961	1580	1202	0.6521	0.7117	0.6806
Inserts	2152	1203	810	0.6414	0.7117	0.7289
Deletes	809	377	392	0.6821	0.6681	0.6750

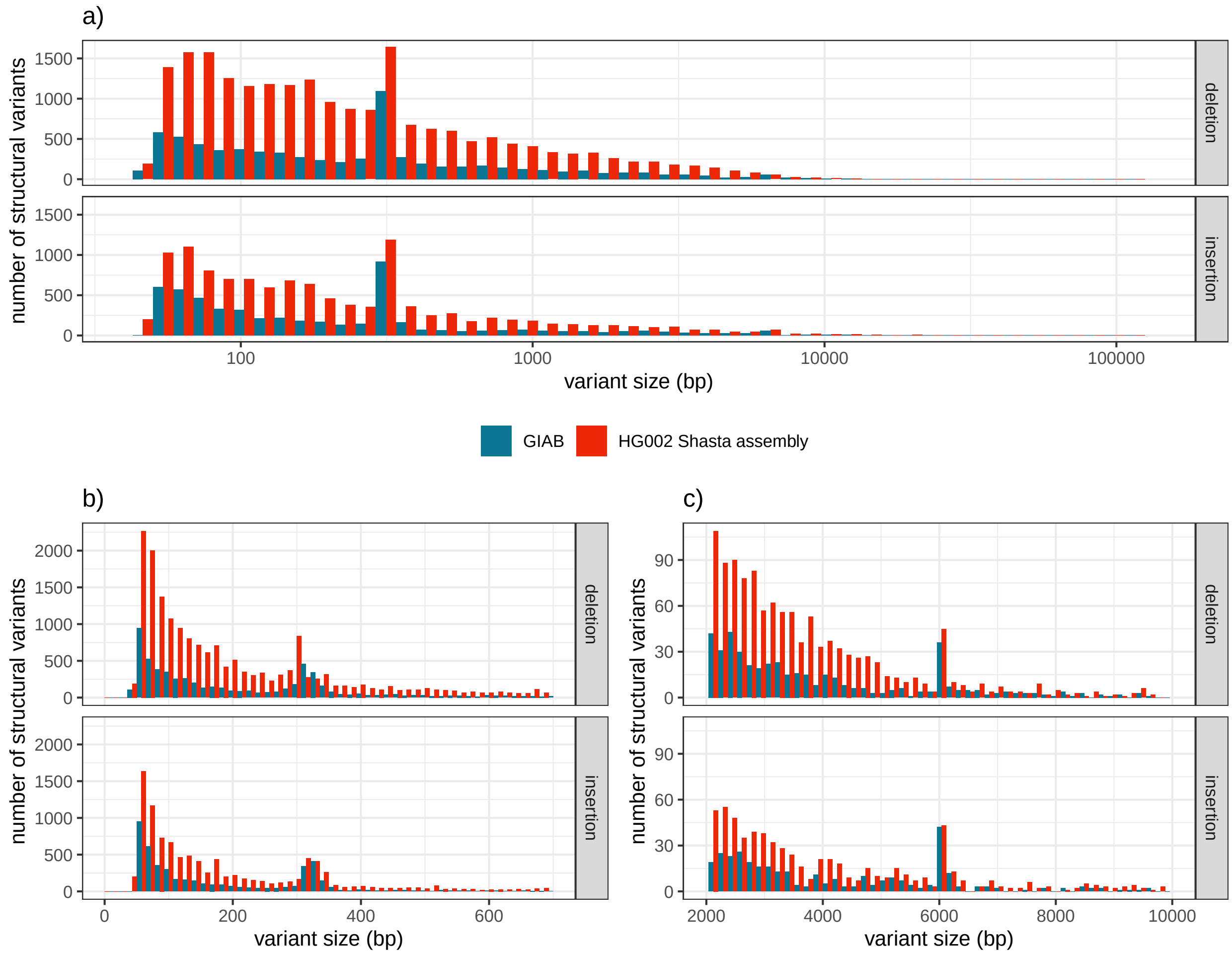

Supplementary Figure 1: Size distribution of structural variants (>50 bp) extracted from the Shasta assembly graph for HG002 and the structural variants in the Genome In A Bottle (GIAB) catalog for the same sample. a) Full size distribution for deletions (top) and insertion (bottom), in log-scale. b) and c) zoom in the two peaks caused by Alu (300 bp) and L1 (6 Kbp) insertion polymorphisms.

Contiguously assembling MHC haplotypes

Supplementary Table 17: CHM13 MHC unpolished Shasta assembly as compared to the nearest matching haplotype in hg38 (GL000251.2)

Assembler	Best Contig	Disagreements	Largest Aligned	Mismatch Rate	Indel Rate
Shasta	62	6	2,788,362	0.00296	0.00399
Canu	tig00589784	5	2,792,139	0.00331	0.00607
Flye	contig_115	6	2,787,570	0.00543	0.01106
wtdbg2	ctg25	32	1,819,753	0.00553	0.00576

Supplementary Table 18: QUAST results for the HG00733 trio-binned maternal reads, using all four assemblers.

Metric	HG00733-Mother			
	Shasta	Wtdbg2	Flye (initial)	Canu
# contigs	1,934	4,028	1,634	877
Total length	2,754,225,214	2,690,619,717	2,791,893,188	2,829,920,708
N50	9,071,623	14,125,235	25,658,831	19,451,828
NG50	7,702,138	10,217,387	23,775,989	16,507,795
# disagreements	705	3,661	6,082	2,161
Genome fraction (%)	90.824	87.373	92.121	92.298
Duplication ratio	0.993	0.996	0.982	0.999
# mismatches per 100 kbp	194.15	287.89	549.61	232.72
# indels per 100 kbp	576.55	859.83	1585.30	724.67
Total aligned length	2,748,135,723	2,650,821,801	2,751,532,754	2,798,797,021
NA50	7,805,090	7,615,651	15,615,208	11,947,316
NGA50	6,339,949	5,584,544	12,833,996	10,085,023

Supplementary Table 19: HG00733 Maternal trio binned MHC unpolished Shasta assembly as compared to the nearest matching haplotype in hg38 (GL000255.1)

Assembler	Best Contig	Disagreements	Largest Aligned	Mismatch Rate	Indel Rate
Shasta	226	0	4,289,729	0.00206	0.00538
Canu	tig00002130	0	4,289,729	0.00182	0.00676
Flye	contig_295	0	4,289,729	0.00579	0.01759
wtdbg2	ctg36	23	1,418,939	0.00592	0.00905

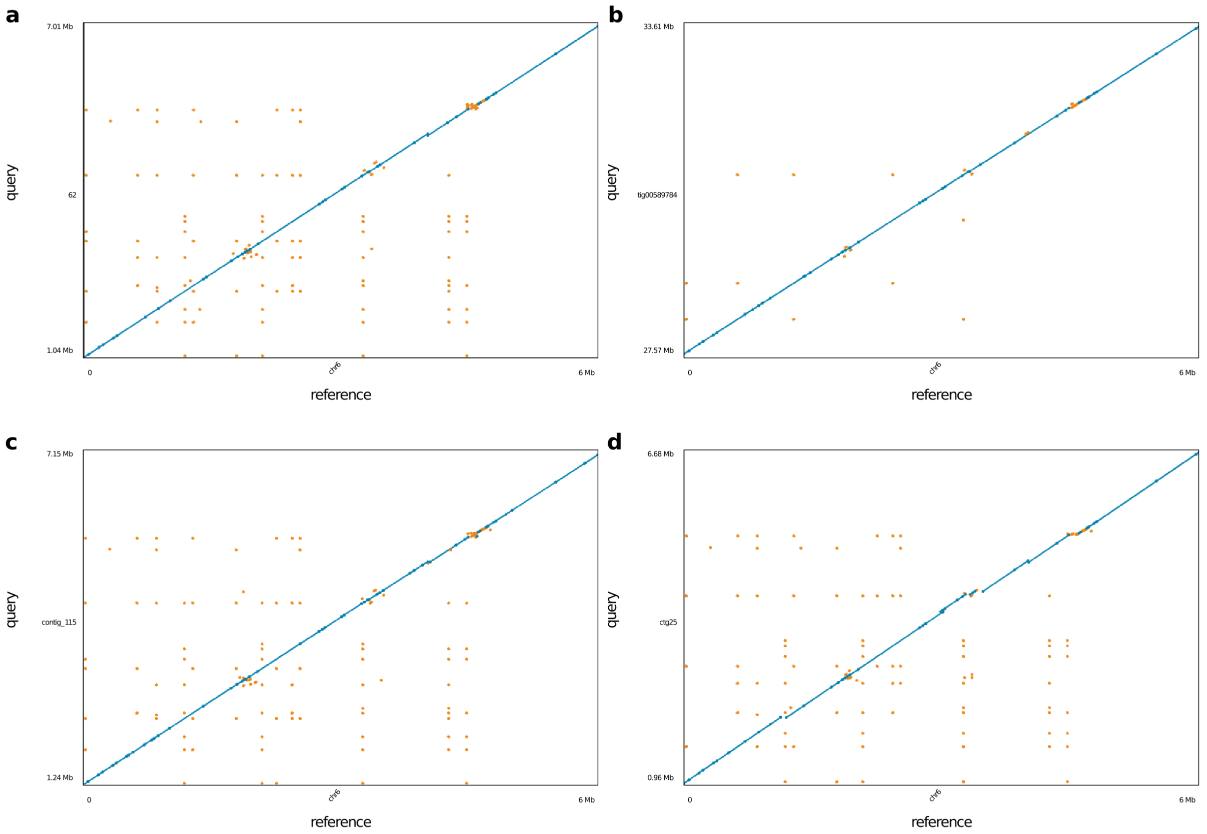

Supplementary Figure 2: Dotplot of unpolished CHM13 MHC assembly vs hg38 chr6:28000000-34000000 for the each of the 4 assemblers tested. **(a)** Shasta **(b)** Canu **(c)** Flye (no native polish) **(d)** wtdbg2. Blue dots represent unique alignments and orange dots represent repetitive alignments.

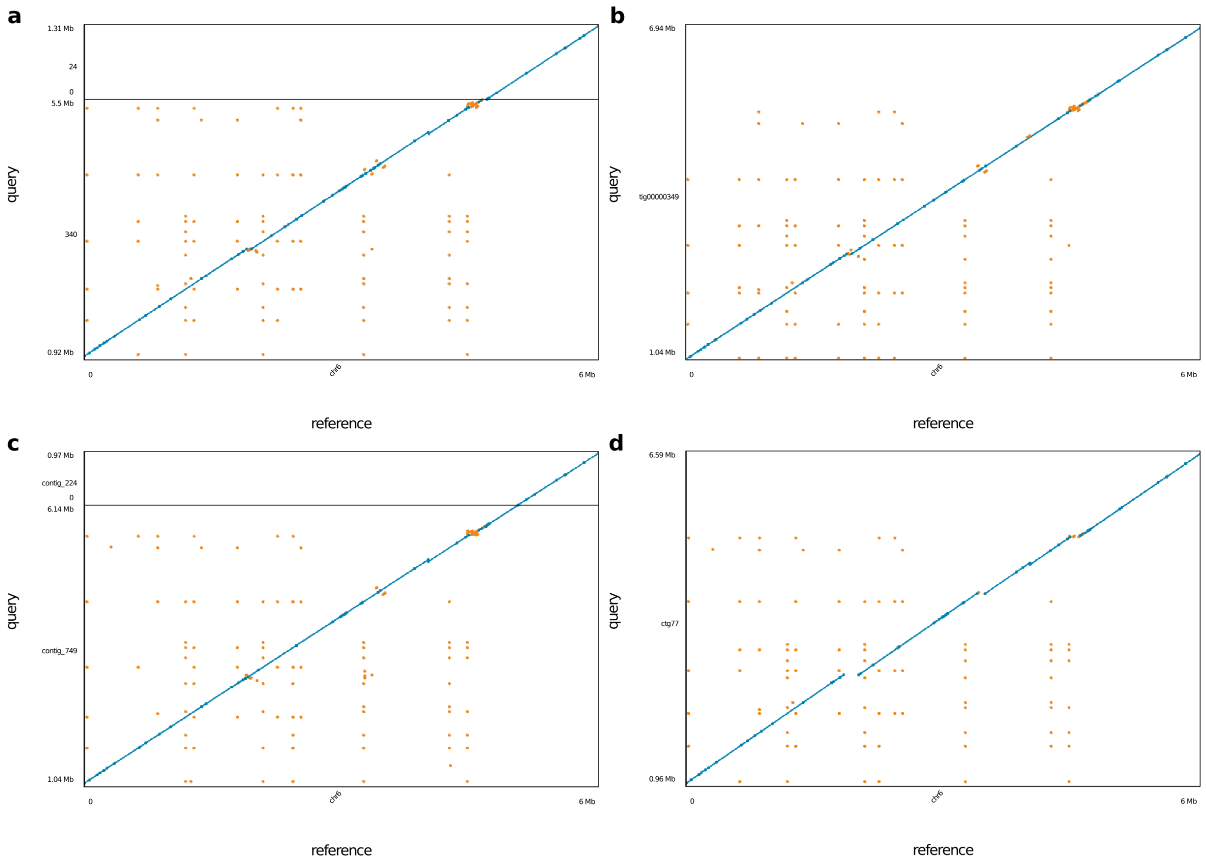

Supplementary Figure 3: Dotplot of unpolished HG00733 diploid MHC assembly vs hg38 chr6:28000000-34000000 for the each of the 4 assemblers tested. **(a)** Shasta **(b)** Canu **(c)** Flye (no native polish) **(d)** wtdbg2. Blue dots represent unique alignments and orange dots represent repetitive alignments.

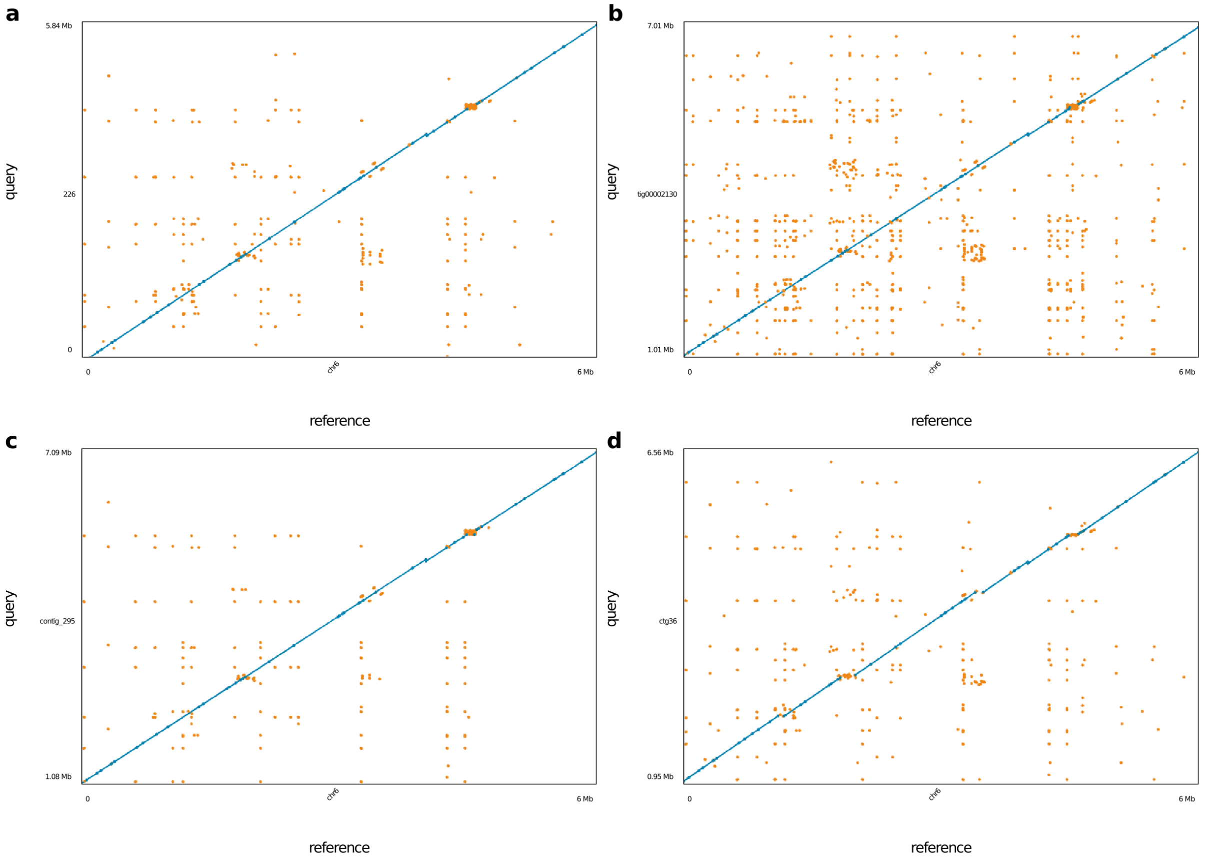

Supplementary Figure 4: Dotplot of unpolished HG00733 maternal haploid MHC assembly vs hg38 chr6:28000000-34000000 for the each of the 4 assemblers tested. **(a)** Shasta **(b)** Canu **(c)** Flye (no native polish) **(d)** wtdbg2. Blue dots represent unique alignments and orange dots represent repetitive alignments.

Deep neural network based polishing achieves QV30 long-read only polishing accuracy

Supplementary Table 20: Base-level accuracies comparing Racon & Medaka and MarginPolish & HELEN pipelines on Shasta assemblies for three samples. Analysis is performed with whole-genome truth sequences.

Sample	Polisher		Percentage Errors			
	Method	Model	Balanced	Identity	Deletion	Insertion
HG002 Guppy 2.3.5	Shasta	Unpolished	0.975%	0.061%	0.849%	0.065%
	Racon	4x	0.665%	0.054%	0.579%	0.032%
	Medaka	r941_flip235	0.393%	0.051%	0.303%	0.039%
	MarginPolish	guppy_ff235	0.372%	0.043%	0.248%	0.081%
	HELEN	rl941_flip235	0.279%	0.038%	0.171%	0.070%
HG00733 Guppy 2.3.5	Shasta	Unpolished	1.062%	0.083%	0.887%	0.093%
	Racon	4x	0.715%	0.080%	0.570%	0.066%
	Medaka	r941_flip235	0.455%	0.075%	0.311%	0.069%
	MarginPolish	guppy_ff235	0.460%	0.063%	0.278%	0.118%
	HELEN	rl941_flip235	0.388%	0.066%	0.202%	0.120%
CHM13 Guppy 2.3.1	Shasta	Unpolished	0.540%	0.039%	0.430%	0.072%
	Racon	4x	0.367%	0.037%	0.199%	0.131%
	Medaka	r941_flip213	0.329%	0.033%	0.037%	0.259%
	MarginPolish	guppy_ff233	0.281%	0.027%	0.071%	0.184%
	HELEN	rl941_flip233	0.206%	0.027%	0.062%	0.117%

Supplementary Table 21: QUAST results for the Shasta assemblies for all samples, post polishing with MarginPolish-HELEN.

Sample	# contigs	Total length	N50	NG50	# mis-assemblies	Genome fraction (%)	# mismatches per 100 kbp	# indels per 100 kbp	Total aligned length	NA50	NGA50
GM24143	2,042	2,802,437,249	23,531,777	19,936,924	970	95.025	128.63	142.77	2,794,379,803	16,323,510	13,840,294
GM24149	2,368	2,816,566,939	20,798,256	17,752,973	990	95.416	130.54	134.60	2,806,847,428	13,174,778	12,128,076
GM24385	1,685	2,819,474,365	23,520,830	20,346,145	960	95.609	127.44	152.17	2,810,951,083	16,200,287	14,315,298
HG00733	1,962	2,800,357,697	24,600,414	21,701,762	877	94.976	126.23	137.92	2,792,792,711	16,156,822	12,971,070
HG01109	2,111	2,820,988,852	21,532,001	18,279,481	1,033	95.564	136.51	140.59	2,811,696,923	13,162,850	12,012,786
HG01243	1,936	2,819,065,027	22,753,128	20,884,160	920	95.521	137.50	143.02	2,810,262,570	16,040,951	14,115,348
HG02055	1,903	2,819,836,390	17,485,643	16,302,857	971	95.592	142.23	162.43	2,810,300,557	13,840,319	12,123,357
HG02080	1,814	2,803,471,776	18,701,305	15,584,440	920	95.045	128.16	134.35	2,794,749,368	12,401,739	11,561,569
HG02723	1,813	2,805,268,038	25,163,327	20,265,678	1,110	95.062	143.30	147.09	2,796,332,696	15,390,923	13,175,818
HG03098	1,790	2,811,295,217	22,571,315	19,620,076	986	95.395	144.36	170.40	2,802,844,336	14,045,283	12,089,849
HG03492	1,811	2,811,690,127	24,629,163	22,891,947	854	95.364	126.61	147.22	2,804,103,412	16,317,390	12,930,516
CHM13	1,186	2,819,245,173	46,206,794	41,255,275	1,107	95.281	136.58	140.38	2,808,536,514	23,540,225	19,532,176

Supplementary Table 22: Base-level accuracies comparing Racon & Medaka and MarginPolish & HELEN pipelines against CHM13 Chromosome-X. The truth Chromosome-X sequence used reflects the most accurate haploid truth sequence available.

Sample	Polisher		Percentage Errors			
	Method	Model	Balanced	Identity	Deletion	Insertion
CHM-13 Chromosome-X	Shasta	Unpolished	0.469%	0.014%	0.404%	0.051%
	Racon	4x	0.313%	0.017%	0.192%	0.104%
	Medaka	r941_flip213	0.110%	0.012%	0.035%	0.063%
	MarginPolish	guppy_ff233	0.215%	0.008%	0.055%	0.153%
	HELEN	rl941_flip233	0.143%	0.007%	0.041%	0.095%
		rl941_flip231	0.064%	0.006%	0.036%	0.022%

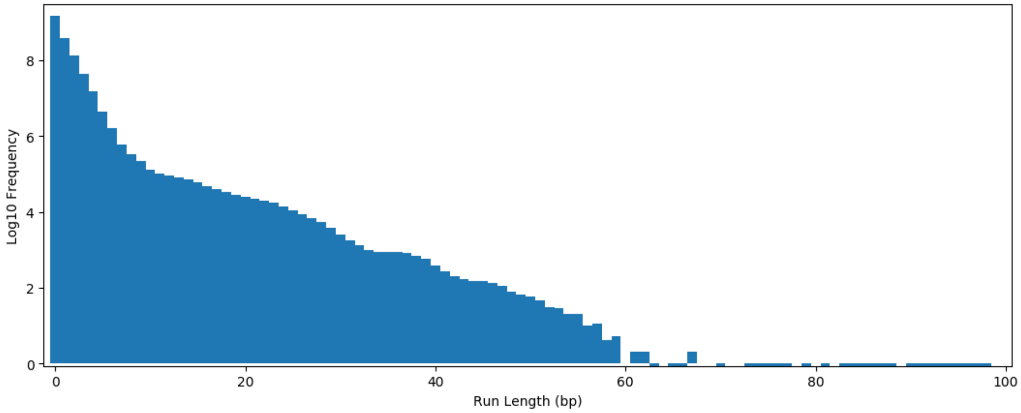

Supplementary Figure 5: Log frequency of each run length as found in the GRCh38 reference for all bases A,C,G,T up to 100bp. Run lengths greater than 15 account for approximately 0.012% of all homopolymer runs in GRCh38.

Supplementary Table 23: Base-level accuracies improvements with MarginPolish and HELEN pipeline on four different assemblers for two samples. Analysis is performed with whole-genome truth sequences.

Sample	Polisher		Percentage Errors			
	Method	Model	Balanced	Identity	Deletion	Insertion
HG00733 Guppy 2.3.5	Shasta	Unpolished	1.062%	0.083%	0.887%	0.093%
	MarginPolish	guppy_ff235	0.460%	0.063%	0.278%	0.118%
	HELEN	rl941_flip235	0.388%	0.066%	0.202%	0.120%
	Wtdbg2	Unpolished	1.217%	0.108%	1.059%	0.051%
	MarginPolish	guppy_ff235	0.538%	0.083%	0.333%	0.122%
	HELEN	rl941_flip235	0.473%	0.089%	0.257%	0.127%
	Canu	Unpolished	1.328%	0.074%	1.224%	0.031%
	MarginPolish	guppy_ff235	0.438%	0.050%	0.290%	0.098%
	HELEN	rl941_flip235	0.355%	0.050%	0.206%	0.099%
	Flye	Unpolished	1.854%	0.089%	0.445%	1.320%
	MarginPolish	guppy_ff235	0.425%	0.062%	0.257%	0.106%
	HELEN	rl941_flip235	0.356%	0.064%	0.183%	0.109%
CHM13 Guppy 2.3.1	Shasta	Unpolished	0.540%	0.039%	0.430%	0.072%
	MarginPolish	guppy_ff233	0.281%	0.027%	0.071%	0.184%
	HELEN	rl941_flip233	0.206%	0.027%	0.062%	0.117%
	Wtdbg2	Unpolished	0.689%	0.068%	0.583%	0.038%
	MarginPolish	guppy_ff233	0.361%	0.049%	0.112%	0.201%
	HELEN	rl941_flip233	0.296%	0.053%	0.115%	0.129%
	Canu	Unpolished	0.705%	0.038%	0.643%	0.024%
	MarginPolish	guppy_ff233	0.255%	0.013%	0.075%	0.168%
	HELEN	rl941_flip233	0.173%	0.012%	0.058%	0.103%
	Flye	Unpolished	2.213%	0.051%	0.448%	1.715%
	MarginPolish	guppy_ff233	0.256%	0.022%	0.058%	0.176%
	HELEN	rl941_flip233	0.185%	0.024%	0.052%	0.109%

Supplementary Table 24: Single-chromosome error rates after polishing with short reads. 10X Chromium reads for sample CHM13 were used to polish via Pilon polishing software. The top half of the table shows the results of three rounds of Pilon, starting from the CHM13 Shasta chrX assembly that had been polished with MarginPolish and HELEN. The bottom half shows the results of three rounds of Pilon, starting from the raw Shasta assembly.

Sample	Assembly	Percentage Errors				Q Scores			
		Balanced	Identity	Deletion	Insertion	Balanced	Identity	Deletion	Insertion
CHM13 ChrX	Shasta (polished)	0.064%	0.006%	0.036%	0.022%	31.92	42.40	34.42	36.51
	Pilon 1x	0.025%	0.004%	0.012%	0.008%	36.06	43.75	39.16	40.75
	Pilon 2x	0.023%	0.004%	0.012%	0.007%	36.29	43.51	39.32	41.34
CHM13 ChrX	Shasta (raw)	0.468%	0.014%	0.404%	0.051%	23.29	38.57	23.94	32.95
	Pilon 1x	0.449%	0.011%	0.395%	0.043%	23.48	39.78	24.03	33.68
	Pilon 2x	0.425%	0.011%	0.373%	0.041%	23.71	39.49	24.29	33.84

Supplementary Table 25: Runtime and cost of two polishing workflows on Amazon Web Services (AWS) platform.

Method	Sample	Minutes	Threads Used	Peak Memory	Instance Type	Instance Cost
Racon (4x)	HG00733	3099	62	574	r5a.24xlarge	$5.42
	GM24385	2342	62	501	r5a.24xlarge	$5.42
	CHM13	3700	62	281	r5a.24xlarge	$5.42
Medaka mini_align	HG00733	611	62	101	c5.18xlarge	$3.06
	GM24385	489	62	115	c5.18xlarge	$3.06
	CHM13	810	60	143	c5.18xlarge	$3.06
Medaka call_consensus	HG00733	8611	62	164	c5n.18xlarge	$3.89
	GM24385	3355	62	150	c5n.18xlarge	$3.89
	CHM13	2532	62	149	c5n.18xlarge	$3.89
MarginPolish	HG00733	680	90	66	m5.metal	$4.61
	HG01109	912	70	57	c5.18xlarge	$3.06
	HG01243	835	70	65	c5.18xlarge	$3.06
	HG02055	733	70	77	c5.18xlarge	$3.06
	HG02080	793	70	64	c5.18xlarge	$3.06
	HG02723	1000	64	60	c5.18xlarge	$3.06
	HG03098	852	70	78	c5.18xlarge	$3.06
	HG03492	777	70	80	c5.18xlarge	$3.06
	GM24385	842	70	66	c5.18xlarge	$3.06
	GM24149	1037	64	103	c5.18xlarge	$3.06
	GM24143	1051	64	84	c5.18xlarge	$3.06
	CHM13	739	70	65	c5.18xlarge	$3.06
HELEN consensus	HG00733	216	8 GPUs	-	p2.8xlarge	$7.20
	HG01109	204	8 GPUs	-	p2.8xlarge	$7.20
	HG01243	233	8 GPUs	-	p2.8xlarge	$7.20
	HG02080	212	8 GPUs	-	p2.8xlarge	$7.20
	HG03098	216	8 GPUs	-	p2.8xlarge	$7.20
	GM24385	208	8 GPUs	-	p2.8xlarge	$7.20
	GM24143	226	8 GPUs	-	p2.8xlarge	$7.20
HELEN stitch	HG00733	59	32	-	p2.8xlarge	$7.20
	HG01109	50	32	-	p2.8xlarge	$7.20
	HG01243	49	32	-	p2.8xlarge	$7.20
	HG02080	54	32	-	p2.8xlarge	$7.20
	HG03098	65	32	-	p2.8xlarge	$7.20
	GM24385	68	32	-	p2.8xlarge	$7.20
	GM24143	62	32	-	p2.8xlarge	$7.20

Supplementary Table 26: Runtime and cost of two polishing workflows run on a 29 Mb contig from the HG00733 Shasta assembly. MarginPolish uses an improved stitch method not used in original runs and Racon was run once instead of four times as was done in the full runs. All runs were configured to use 32 CPUs, except for the GPU runs which were performed with 16 CPUs and 1 GPU (Tesla P100).

**Application**	**Runtimes**	**Avg Runtime**
MarginPolish	16.6	16.46
	16.47	
	16.31	
HELEN consensus (CPU)	97.46	95.86
	95.55	
	94.56	
HELEN consensus (GPU)	1.63	1.67
	1.72	
	1.65	
HELEN stitch	0.76	0.78
	0.78	
	0.80	
Racon 1x	52.00	52.04
	52.15	
	51.98	
mini_align	3.01	3.00
	3.00	
	2.98	
Medaka (CPU)	17.26	17.01
	16.78	
	16.98	
Medaka consensus (GPU)	10.55	10.62
	10.73	
	10.57	
Medaka stitch (GPU)	0.68	0.68
	0.68	
	0.68	

Long-read assemblies contain nearly all human coding genes

Supplementary Table 27: Transcript-level analysis with Comparative Annotation Toolkit (CAT) of MarginPolish & HELEN and Racon & Medaka on three samples from Shasta assemblies.

Metric		HG002		HG00733		CHM13	
		HELEN	MEDAKA	HELEN	MEDAKA	HELEN	MEDAKA
Transcripts Found	Total	83093	83105	83002	82928	82833	82807
	Percent	99.536	99.551	99.427	99.339	99.225	99.194
Full mRNA Coverage	Total	25721	20367	28612	26573	40132	38081
	Percent	30.811	24.397	34.274	31.832	48.074	45.617
Full CDS Coverage	Total	41396	36248	45104	43956	53089	52297
	Percent	49.588	43.421	54.030	52.655	63.595	62.646
Transcripts With Frameshift	Total	35339	40783	31333	32647	23261	24441
	Percent	42.332	48.854	37.534	39.108	27.864	29.278
Transcripts With Original Introns	Total	76880	76883	76618	76463	76807	76803
	Percent	92.094	92.098	91.780	91.594	92.006	92.002
Transcripts With Full CDS Coverage	Total	41396	36248	45104	43956	53089	52297
	Percent	49.588	43.421	54.030	52.655	63.595	62.646
Transcripts With Full CDS Coverage And No Frameshifts	Total	41245	36158	44982	43860	52966	52160
	Percent	49.407	43.313	53.884	52.540	63.448	62.482
Transcripts With Full CDS Coverage And No Frameshifts And Original Introns	Total	41021	35952	44692	43546	52616	51807
	Percent	49.139	43.067	53.536	52.163	63.028	62.059

Supplementary Table 28: Gene-level analysis with Comparative Annotation Toolkit (CAT) of MarginPolish & HELEN and Racon & Medaka on three samples from Shasta assemblies.

Metric		HG002		HG00733		CHM13	
		HELEN	MEDAKA	HELEN	MEDAKA	HELEN	MEDAKA
Genes Found	Total	19536	19531	19537	19511	19505	19490
	Percent	99.268	99.243	99.273	99.141	99.111	99.035
Genes With Frameshift	Total	10933	12165	9941	10081	7300	7564
	Percent	55.554	61.814	50.513	51.225	37.093	38.435
Genes With Original Introns	Total	18212	18198	18151	18113	18217	18202
	Percent	92.541	92.47	92.231	92.038	92.566	92.49
Genes With Full CDS Coverage	Total	11070	10066	11812	11756	13648	13534
	Percent	56.25	51.148	60.02	59.736	69.35	68.77
Genes With Full CDS Coverage And No Frameshifts	Total	12454	11570	13127	13081	14625	14562
	Percent	63.283	58.791	66.702	66.468	74.314	73.994
Genes With Full CDS Coverage And No Frameshifts And Original Introns	Total	12422	11539	13098	13042	14603	14531
	Percent	63.12	58.633	66.555	66.27	74.202	73.836
Missing Genes	Total	144	149	143	169	175	190
	Percent	0.732	0.757	0.727	0.859	0.889	0.965

Supplementary Table 29: Transcript-level analysis with Comparative Annotation Toolkit (CAT) of four HG00733 assemblies polished with MarginPolish and HELEN.

Metric		HG00733			
		Flye HELEN	Canu HELEN	Wtdbg2 HELEN	Shasta HELEN
Transcripts Found	Total	83267	83334	81484	82974
	Percent	99.745	99.825	97.609	99.394
Full mRNA Coverage	Total	33078	28488	28889	30378
	Percent	39.624	34.126	34.606	36.390
Full CDS Coverage	Total	41396	44877	45321	46965
	Percent	59.754	53.758	54.290	56.259
Transcripts With Frameshift	Total	27293	32230	29525	29657
	Percent	32.694	38.608	35.368	35.526
Transcripts With Original Introns	Total	77412	77583	74683	76613
	Percent	92.731	92.936	89.462	91.774
Transcripts with Full CDS Coverage	Total	49883	44877	45321	46965
	Percent	59.754	53.758	54.290	56.259
Transcripts with Full CDS Coverage And No Frameshifts	Total	49766	44737	45217	46802
	Percent	59.614	53.590	54.165	56.064
Transcripts with Full CDS Coverage And No Frameshifts And Original Introns	Total	49459	44412	44924	46505
	Percent	59.247	53.201	53.814	55.708

Supplementary Table 30: Gene-level analysis with Comparative Annotation Toolkit (CAT) of four HG00733 assemblies polished with MarginPolish and HELEN

Metric		HG00733			
		Flye HELEN	Canu HELEN	Wtdbg2 HELEN	Shasta HELEN
Genes Found	Total	19563	19629	19174	19528
	Percent	99.405	99.741	97.429	99.228
Genes With Frameshift	Total	8698	10160	9323	9464
	Percent	44.197	51.626	47.373	48.089
Genes With Original Introns	Total	18345	18460	17709	18154
	Percent	93.216	93.801	89.985	92.246
Genes With Full CDS Coverage	Total	12966	11889	11817	12207
	Percent	65.884	60.412	60.046	62.027
Genes With Full CDS Coverage And No Frameshifts	Total	14145	13221	13047	13419
	Percent	71.875	67.18	66.296	68.186
Genes With Full CDS Coverage And No Frameshifts And Original Introns	Total	14124	13193	13017	13396
	Percent	71.768	67.038	66.143	68.069
Missing Genes	Total	117	51	506	152
	Percent	0.595	0.259	2.571	0.772

Supplementary Table 31: BUSCO results of three samples using two polishing workflows on Shasta assemblies.

Sample	Metric	Shasta MarginPolish HELEN	Shasta Racon (4x) Medaka
HG00733	Complete BUSCOs (C)	87.20%	87.10%
	Complete and single-copy BUSCOs (S)	84.20%	83.80%
	Complete and duplicated BUSCOs (D)	3.00%	3.30%
	Fragmented BUSCOs (F)	4.60%	5.30%
	Missing BUSCOs (M)	8.20%	7.60%
HG002	Complete BUSCOs (C)	89.40%	88.80%
	Complete and single-copy BUSCOs (S)	84.80%	85.80%
	Complete and duplicated BUSCOs (D)	4.60%	3.00%
	Fragmented BUSCOs (F)	3.60%	4.30%
	Missing BUSCOs (M)	7.00%	6.90%
CHM13	Complete BUSCOs (C)	86.50%	86.80%
	Complete and single-copy BUSCOs (S)	82.50%	82.80%
	Complete and duplicated BUSCOs (D)	4.00%	4.00%
	Fragmented BUSCOs (F)	5.90%	5.30%
	Missing BUSCOs (M)	7.60%	7.90%

Supplementary Table 32: BUSCO results for four assemblers on HG00733, post polishing with MarginPolish and HELEN.

Metric	HG00733			
	Flye	Canu	Wtdbg2	Shasta
Complete BUSCOs (C)	87.50%	89.80%	85.80%	87.20%
Complete and single-copy BUSCOs (S)	84.50%	86.80%	82.20%	84.20%
Complete and duplicated BUSCOs (D)	3.00%	3.00%	3.60%	3.00%
Fragmented BUSCOs (F)	5.30%	3.00%	6.30%	4.60%
Missing BUSCOs (M)	7.20%	7.20%	7.90%	8.20%

Comparing to a PacBio HiFi Assembly

Supplementary Table 33: CHM13 QUAST results for Shasta, MarginPolish, HELEN and PacBio HiFi assembly. Stratified disagreement counts were added after manual determination.

Metric	CHM13	
	Nanopore Shasta MarginPolish, HELEN	PacBio-HiFi Canu Racon
# contigs	1622	5206
Total length	2819245173	3031026325
N50	46206794	29522819
NG50	41255275	29092230
# disagreements	1107	8666
# disagreements outside Centromeres	801	2999
# disagreements outside centromeres and Seg Dups	314	893
Genome fraction (%)	95.281	97.030
# mismatches per 100 kbp	136.58	274.84
# indels per 100 kbp	140.38	32.99
Total aligned length	2808536514	2954558720
NA50	23540225	20440378
NGA50	19532176	20029136

Supplementary Table 34: Disagreement count in the intersection of the assemblies between the PacBio-HiFi and the Shasta assembly of CHM13. Total Disagreements is all disagreements found in 100bp before windows before taking the intersection, note it is very close to that reported by QUAST. Consensus disagreements: Disagreements in the intersection of the four assemblies.

Sample	Assembler	Total disagreements	Consensus disagreements
CHM13	PacBio-HiFi	8469	594
	Shasta	1073	380

Supplementary Table 35: CHM13 Chromosome-X error rate analysis with Pomoxis for Shasta, MarginPolish, HELEN, and PacBio HiFi assembly.

Sample	Sequencing Platform	Method		Percentage errors			
		Assembler	Polisher	Balanced	Identity	Deletion	Insertion
CHM13 Chr-X	PacBio HiFi	Canu	Racon	0.008%	0.001%	0.004%	0.003%
	Nanopore	Shasta	MarginPolish & HELEN	0.064%	0.006%	0.036%	0.022%

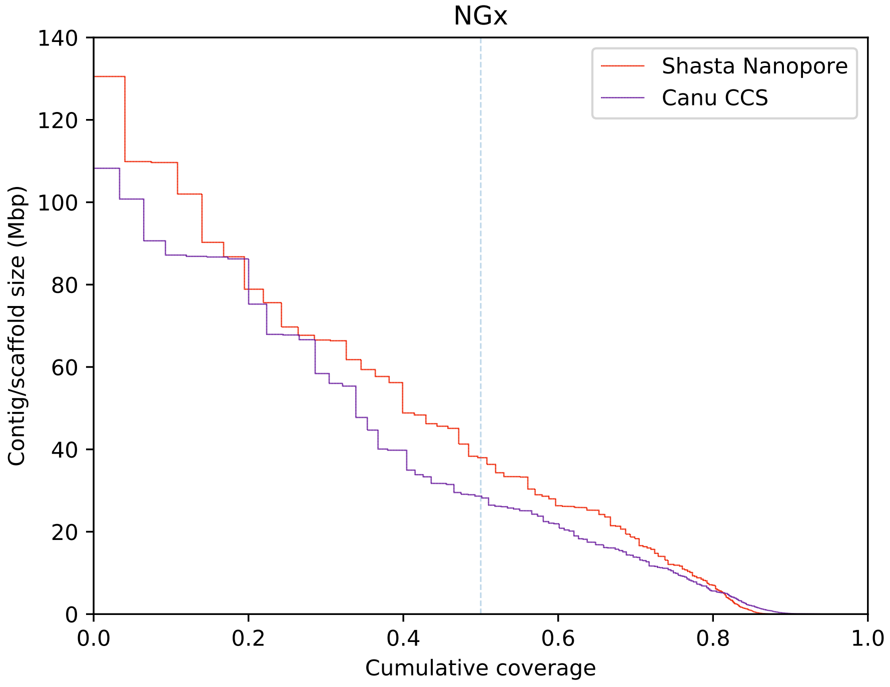

Supplementary Figure 6: Contig NGx for CHM13 Shasta-HELEN nanopore assembly vs Canu CCS (HiFi) assembly

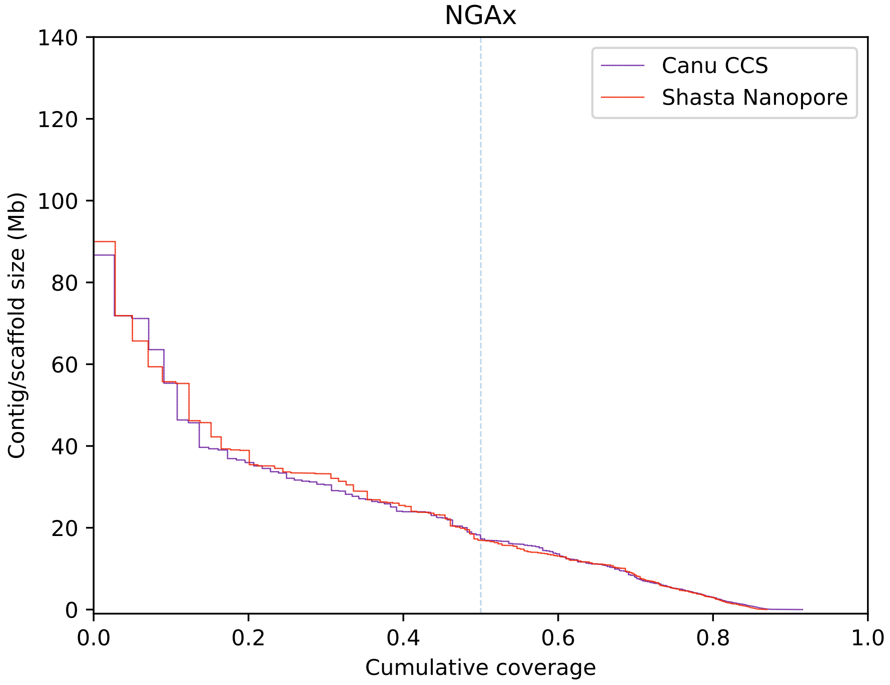

Supplementary Figure 7: Contig NGAx for CHM13 Shasta-HELEN nanopore assembly vs Canu CCS (HiFi) assembly

Assembling, polishing and scaffolding 11 human genomes at near chromosome scale

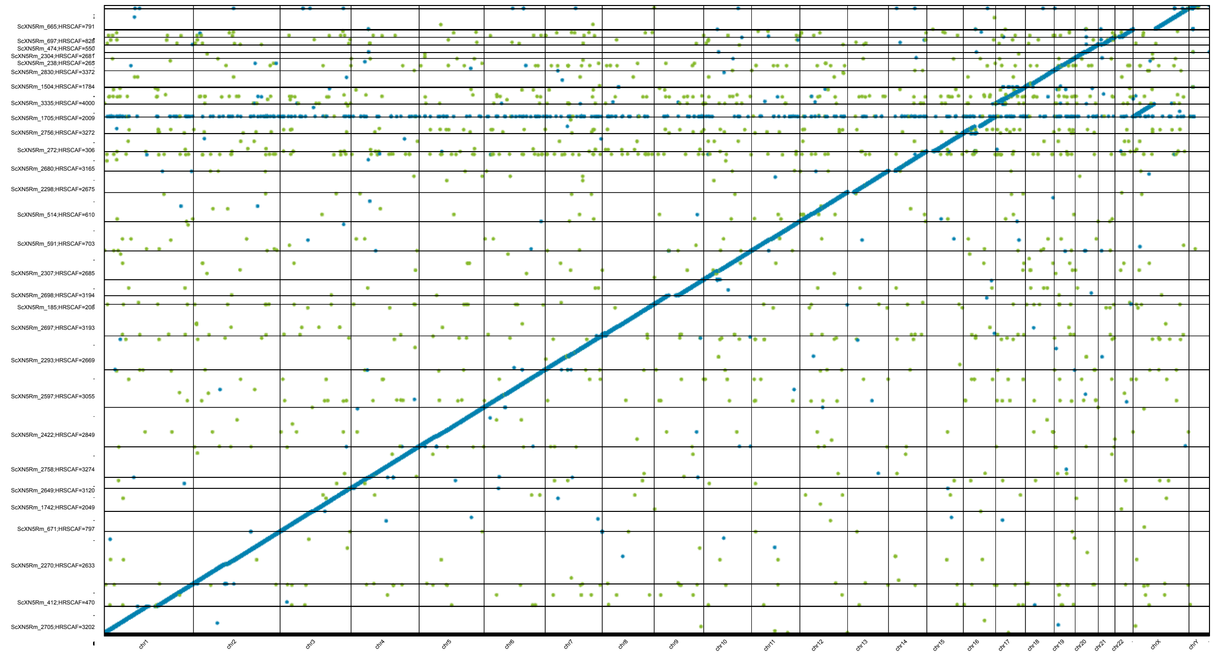

Supplementary Figure 8: Dotplot for the scaffolded HG002 assembly, aligned with GRCh38. Blue dots represent unique alignments and orange dots represent repetitive alignments.

Supplementary Table 36: QUAST results for all 11 Shasta assemblies scaffolded with HiRise, post polishing with MarginPolish-HELEN

Sample	# contigs	Total length	N50	NG50	# mis-assemblies	# scaffold gap extensive mis-assembies	Genome fraction (%)	# mismatches per 100 kbp	# indels per 100 kbp	Total aligned length	NA50	NGA50
GM24143	1,184	2,802,523,049	129,960,437	128,216,303	1,466	4	95.027	128.28	142.79	2,792,775,664	20,657,530	16,966,477
GM24149	1,323	2,816,683,224	129,643,816	128,275,807	1,530	11	95.417	130.24	134.58	2,804,735,382	18,446,390	15,435,923
GM24385	1,019	2,819,527,260	118,169,209	102,591,941	1,335	6	95.606	127.19	152.25	2,809,570,528	22,369,161	16,601,924
HG00733	1,056	2,800,455,909	129,857,865	118,785,172	1,337	8	94.974	126.16	138.09	2,791,610,554	22,141,375	17,570,210
HG01109	1,156	2,821,098,626	130,282,751	130,166,418	1,529	5	95.559	136.73	140.63	2,809,413,640	19,932,703	17,228,023
HG01243	1,006	2,819,162,443	128,571,344	118,762,399	1,381	7	95.517	137.47	143.03	2,808,041,766	22,146,722	17,559,055
HG02055	977	2,819,933,140	130,184,428	128,180,737	1,387	8	95.587	141.91	162.46	2,809,195,864	21,057,279	18,446,049
HG02080	934	2,803,570,658	129,931,575	128,451,196	1,470	9	95.041	127.98	134.36	2,793,854,132	20,418,609	16,379,851
HG02723	982	2,805,356,030	130,365,062	128,975,828	1,499	9	95.06	143.45	147.13	2,794,747,200	20,232,566	17,865,825
HG03098	926	2,811,385,538	130,040,472	128,535,908	1,439	4	95.391	144.36	170.40	2,801,774,564	22,165,948	17,439,948
HG03492	901	2,811,782,250	130,277,907	100,251,163	1,381	7	95.362	126.54	147.23	2,803,106,787	20,001,587	16,836,756
