# Supplementary material for: Nanopore sequencing and the Shasta toolkit enable efficient de novo assembly of eleven human genomes
Source: Nat Biotechnol. 2020 May 4;38(9):1044–53. doi: 10.1038/s41587-020-0503-6 (PMC7483855; doi:10.1038/s41587-020-0503-6)
Supplement: Supplementary file 2 — Reporting Summary [file 41587_2020_503_MOESM2_ESM.pdf]

natureresearch

Corresponding author(s): Paten B, Jain M, Carnevali P

Last updated by author(s): Mar 12, 2020

Reporting Summary

Nature Research wishes to improve the reproducibility of the work that we publish. This form provides structure for consistency and transparency in reporting. For further information on Nature Research policies, see Authors & Referees and the Editorial Policy Checklist.

Statistics

For all statistical analyses, confirm that the following items are present in the figure legend, table legend, main text, or Methods section.

n/a	Confirmed	
☐	☒	The exact sample size (*n*) for each experimental group/condition, given as a discrete number and unit of measurement
☒	☐	A statement on whether measurements were taken from distinct samples or whether the same sample was measured repeatedly
☒	☐	The statistical test(s) used AND whether they are one- or two-sided<br>*Only common tests should be described solely by name; describe more complex techniques in the Methods section.*
☒	☐	A description of all covariates tested
☒	☐	A description of any assumptions or corrections, such as tests of normality and adjustment for multiple comparisons
☐	☒	A full description of the statistical parameters including central tendency (e.g. means) or other basic estimates (e.g. regression coefficient) AND variation (e.g. standard deviation) or associated estimates of uncertainty (e.g. confidence intervals)
☒	☐	For null hypothesis testing, the test statistic (e.g. *F*, *t*, *r*) with confidence intervals, effect sizes, degrees of freedom and *P* value noted<br>*Give P values as exact values whenever suitable.*
☐	☒	For Bayesian analysis, information on the choice of priors and Markov chain Monte Carlo settings
☒	☐	For hierarchical and complex designs, identification of the appropriate level for tests and full reporting of outcomes
☒	☐	Estimates of effect sizes (e.g. Cohen's *d*, Pearson's *r*), indicating how they were calculated

Our web collection on statistics for biologists contains articles on many of the points above.

Software and code

Policy information about availability of computer code

Data collection

We used Nanopore Technologies MinKnow software that comes with the sequencing device by default. The Guppy basecaller model we used was Guppy 2.3.5.

Data analysis

The online methods and supplementary notes sections describe all available software and the commands we used for data analysis.

Base calling: Oxford Nanopore Technology provided Guppy basecaller v2.3.5 (commercial product)
Shasta: https://github.com/chanzuckerberg/shasta (MIT License)
Canu: https://github.com/marbl/canu (GNU General Public License, version 2)
WTDBG2: https://github.com/ruanjue/wtdbg2 (GPL-3.0 License)
Flye: https://github.com/fenderglass/Flye (BSD-3-Clause License)
Racon 4x: https://github.com/rlorigro/nanopore_assembly_and_polishing_assessment (MIT License)
Medaka: https://github.com/nanoporetech/medaka (MPL-2.0 License)
Pomoxis (mini_align and assess_assembly): https://github.com/nanoporetech/pomoxis (MPL-2.0 License)
Minimap2: https://github.com/lh3/minimap2 (MIT License)
MarginPolish: https://github.com/UCSC-nanopore-cgl/MarginPolish (MIT License)
HELEN: https://github.com/kishwarshafin/helen (MIT License)
CAT: https://github.com/ComparativeGenomicsToolkit/Comparative-Annotation-Toolkit (Apache-2.0 License)
Read alignment identity: https://github.com/rlorigro/nanopore_assembly_and_polishing_assessment (MIT License)
QUAST: https://github.com/rlorigro/quast (GNU General Public License, Version 2)
misassembly stats: https://github.com/kishwarshafin/helen/blob/master/modules/python/helper/quast_sv_extractor.py (MIT License)
Run-length confusion matrix: https://github.com/rlorigro/runlength_analysis/ (MIT License)
Runtime and cost analysis: https://github.com/rlorigro/TaskManager (MIT License)
BAC analysis: https://github.com/skoren/bacValidation (Public domain)

For manuscripts utilizing custom algorithms or software that are central to the research but not yet described in published literature, software must be made available to editors/reviewers. We strongly encourage code deposition in a community repository (e.g. GitHub). See the Nature Research guidelines for submitting code & software for further information.

Data

Policy information about availability of data

All manuscripts must include a data availability statement. This statement should provide the following information, where applicable:

- Accession codes, unique identifiers, or web links for publicly available datasets
- A list of figures that have associated raw data
- A description of any restrictions on data availability

Sequence data including raw signal files (FAST5), base-calls (FASTQ), Illumina Hi-C data (FASTQ), are publicly hosted on Amazon Web Services Public Datasets program and available for download via GitHub here: https://github.com/human-pangenomics/hpgp-data

Field-specific reporting

Please select the one below that is the best fit for your research. If you are not sure, read the appropriate sections before making your selection.

☒ Life sciences ☐ Behavioural & social sciences ☐ Ecological, evolutionary & environmental sciences

For a reference copy of the document with all sections, see nature.com/documents/nr-reporting-summary-flat.pdf

Life sciences study design

All studies must disclose on these points even when the disclosure is negative.

Sample size	No sample size calculation was performed. Cells were grown in batches and aliquoted into 50 million sized pellets. We chose 50M sized cell pellets to ensure we could isolate sufficient DNA for the experimental protocols. The 50M size was selected based on prior literature (Jain et al. NBT 2018).
Data exclusions	No data were excluded from the analyses.
Replication	We performed three replicate experiments for data generation. The replicates were consistent in data quality, as demonstrated in Figure 1.
Randomization	No randomization was performed by group; however, this was not relevant to our study as data from groups was pooled and analyzed together.
Blinding	Investigators were not blind to group allocation during experiments, as all data from groups was pooled together.

Reporting for specific materials, systems and methods

We require information from authors about some types of materials, experimental systems and methods used in many studies. Here, indicate whether each material, system or method listed is relevant to your study. If you are not sure if a list item applies to your research, read the appropriate section before selecting a response.

Materials & experimental systems

n/a	Involved in the study	
☒	☐	Antibodies
☐	☒	Eukaryotic cell lines
☒	☐	Palaeontology
☒	☐	Animals and other organisms
☒	☐	Human research participants
☒	☐	Clinical data

Methods

n/a	Involved in the study	
☒	☐	ChIP-seq
☒	☐	Flow cytometry
☒	☐	MRI-based neuroimaging

Eukaryotic cell lines

Policy information about cell lines

Cell line source(s)	Coriell Institute. Cell lines: HG002, HG003, HG004, HG02055, HG02080, HG03492, HG00733, HG03098, HG01243, HG02723, HG01109
Authentication	The cell lines used were not authenticated.
Mycoplasma contamination	The cell lines used were not tested for mycoplasma contamination.
Commonly misidentified lines (See ICLAC register)	The cell lines used are not in the register of commonly misidentified lines.
